# Prediction and potential risk factors for electronic cigarette use behaviors among adolescents: a pilot study in Chiayi, Taiwan

**DOI:** 10.3389/fpubh.2023.1140615

**Published:** 2023-06-15

**Authors:** Ping-I Liu, Ming-Nan Lin, Pei-Shan Ho, Jui-Hsiu Tsai, Ya-Hui Yang, Ke-Fei Wu, Hung-Yi Chuang

**Affiliations:** ^1^Department of Family Medicine, Dalin Tzu Chi Hospital, Buddhist Tzu Chi Medical Foundation, Chia-Yi, Taiwan; ^2^Department of Geriatric Medicine, Dalin Tzu Chi Hospital, Buddhist Tzu Chi Medical Foundation, Chia-Yi, Taiwan; ^3^School of Medicine, Tzu Chi University, Hualien, Taiwan; ^4^Department of Medical Research, Kaohsiung Medical University Hospital, Kaohsiung Medical University, Kaohsiung, Taiwan; ^5^Department of Oral Hygiene, College of Dental Medicine, Kaohsiung Medical University, Kaohsiung, Taiwan; ^6^School of Dentistry, College of Dental Medicine, Kaohsiung Medical University, Kaohsiung, Taiwan; ^7^Department of Psychiatry, Dalin Tzu Chi Hospital, Buddhist Tzu Chi Medical Foundation, Chia-Yi, Taiwan; ^8^Department of Health-Business Administration, Fooyin University, Kaohsiung, Taiwan; ^9^Department of Business Management, National Taichung University of Science and Technology, Taichung, Taiwan; ^10^Ph.D. Program in Environmental and Occupational Medicine, College of Medicine, and Research Center for Precision Environmental Medicine, Kaohsiung Medical University, Kaohsiung, Taiwan; ^11^Department of Environmental and Occupational Medicine, Kaohsiung Medical University Hospital, Kaohsiung Medical University, Kaohsiung, Taiwan

**Keywords:** electronic cigarette, adolescent, prediction, risk factors, tobacco

## Abstract

**Introduction:**

Electronic cigarette (e-cigarette) use among adolescents has become increasingly common; therefore, effectively reducing adolescent e-cigarette use is an urgent issue. We aimed to predict and identify potential factors related to adolescent e-cigarette use behaviors.

**Methods:**

This cross-sectional study was conducted using anonymous questionnaires given to Taiwanese high school students in 2020. Approximately 1,289 adolescent students completed questions on e-cigarette use, personal characteristics, family environment, and substances used. We performed multivariate logistic regression analyses to assess the model’s predictive performance in terms of the area under the receiver operating characteristic curve.

**Results:**

We found that 9.3% of adolescent students used e-cigarettes. Tobacco smoking, close friends’ reactions to e-cigarette use, and the use of other substances were independent risk factors for adolescent e-cigarette use. Furthermore, relative to tobacco nonuse, tobacco use and tobacco smoking dependence had odds ratios of 76.49 and 113.81, respectively. The predictive accuracy of adolescent e-cigarette use from personal characteristics, family environment, and substance use status was 73.13, 75.91, and 93.80%, respectively.

**Conclusion:**

The present study highlights the need for early prevention of e-cigarette use among adolescents, particularly those with a history of using tobacco and other substances and those who have close friends with positive attitudes towards e-cigarette use.

## Introduction

Electronic cigarettes (e-cigarettes), battery-powered nicotine delivery systems, have been available for purchase since 2005 (1, 2). E-cigarettes were initially developed to help chain smokers quit tobacco smoking (3). Although e-cigarettes’ safety and health effects remain dangerous or lethal particularly by children and adolescents (2, 4), they are thus popular among adolescents and young adults. This is because e-cigarettes are available in numerous flavors, which lead potential users to feel curious about trying them; can be fashionable, stimulating, and a statement of rebellion against conventional values; and may increase positive moods (4–7). Specifically, in the United States, the prevalence of adolescent e-cigarette use increased from 4.2% in 2008 to 16.0% in 2015. In Taiwan, adolescent e-cigarette uses more than doubled from 2.1% in 2014 to 4.5% in 2017 (8, 9). The growing popularity of e-cigarettes among adolescents has raised concerns from policymakers and tobacco control advocates (10).

**Figure 1:**
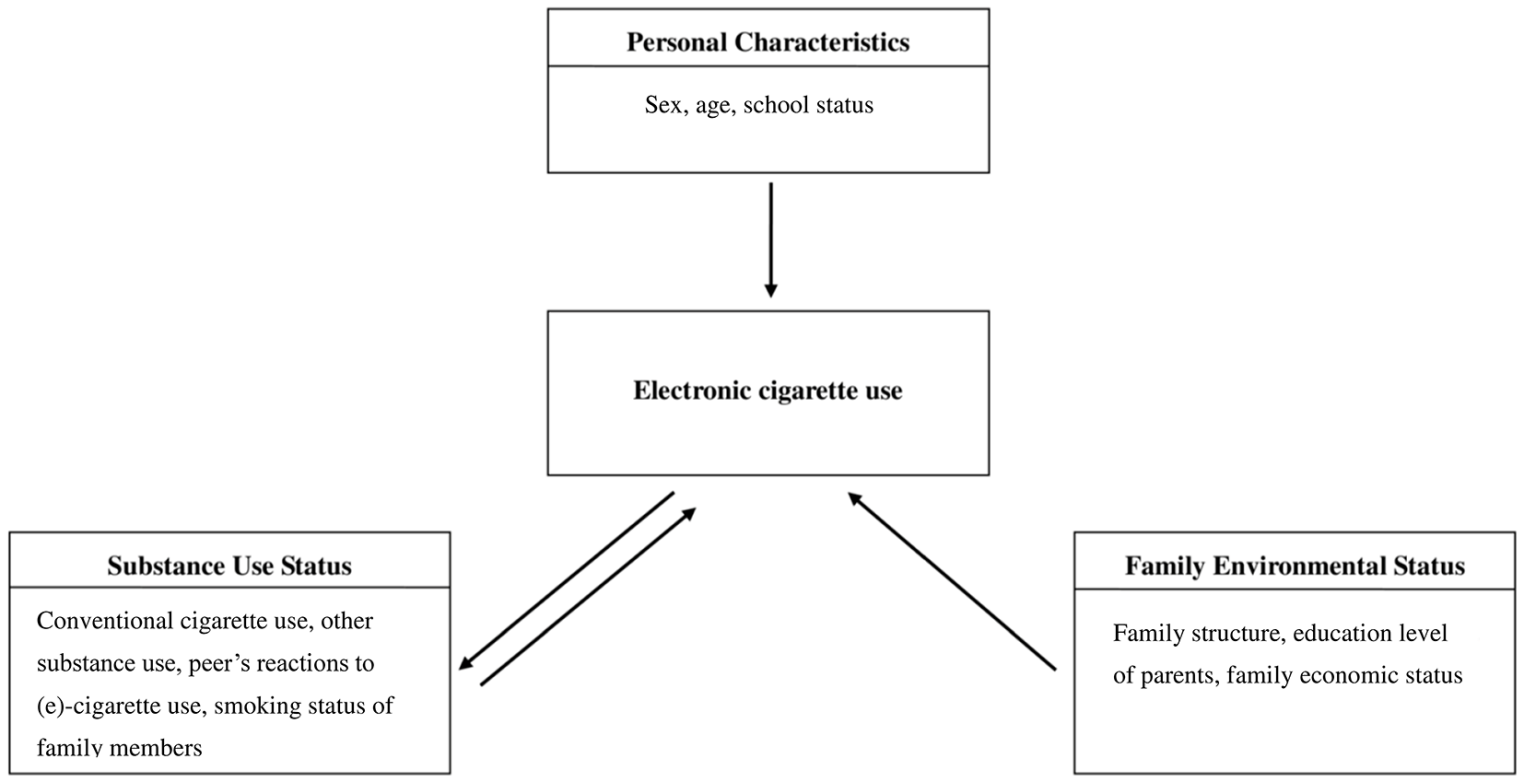
Model of risk factors for electronic cigarette use in adolescents.

To date, several risk factors for e-cigarette use have been identified, including personal and sociodemographic characteristics (e.g., age, sex, ethnicity, and school status); substance use (e.g., tobacco); and social factors (close friends to e-cigarette use), particularly with regard to the family (e.g., smoking status of family members, family structure, education level of parents, allowance, and family economic status; Figure 1) (11–26). Most evidence related to adolescent e-cigarette use in Taiwan focused on the prevalence (10, 24, 25, 27–29); however, few studies assessed the risk factors for adolescent e-cigarette use in Taiwan (24–26). We therefore tended to re-identify these risk factors and develop a predictive model. In addition, we examined whether tobacco smoking initiation is associated with the risk of adolescent e-cigarette use (11, 20, 23, 25, 26) and further whether tobacco smoking dependence level influences e-cigarette use among adolescents.

We conducted an anonymous cross-sectional survey of Taiwanese high school students to identify the risk factors for e-cigarette use. Our findings aid tailored interventions against e-cigarette use among adolescents. Furthermore, we investigated whether tobacco smoking dependence level affected e-cigarette use among adolescents.

## Methods

### Study design and participants

This cross-sectional study distributed anonymous pen-and-paper questionnaires to 12–18-year-old high school students in Chiayi, Taiwan, between January and April 2020. We classified the 58 high schools distributed across Chiayi City and Chiayi County into two subgroups by grade: grades 7–9 (junior high school) and 10–12 (senior high school and vocational high school). We applied multi-level sampling methods for sample selection. First level was administrative areas. There were two administrative areas–Chiayi city and Chiayi country–in Chiayi, Taiwan. And second level was school subgroup. We classified into three subgroup high schools by the grade, which include grades 7–9 (junior high school), grades 10–12 (senior high school), and grades 10–12 (vocational high school). The schools were selected from each subgroup high school by probability proportionate to size sampling, so we selected 16 high schools (including 8 junior high schools, 4 senior high schools, and 4 vocational high schools) among 58 high schools in Chiayi areas.

### Procedures and measures

Participants completed the self-administered questionnaire in their classrooms. To ensure that each participant responded willingly and truthfully to the questionnaire, no school staff members were present when the survey was completed. The data were collected by specialists and trained residents from the Dalin Tzu Chi Hospital family medicine department, who provided standardised instructions to the participants.

The questionnaire items covered the following three dimensions: (1) personal characteristics, namely sex, age, and school status (senior high school, junior high school, and vocational school); (2) family environment status, namely family structure, education levels of parents, parents’ ethnicities, allowance, and family economic status; and (3) substance use status, including tobacco use, use of other substances, close friends’ attitudes to tobacco smoking, e-cigarette use, and smoking status of family members. Participants who responded “yes” to “Have you ever used tobacco cigarettes or cigars?” were considered to be tobacco users. If a participant was a cigarette user who had smoked a cigarette in the past month and used ≥100 cigarettes in their lifetime, their level of dependence on tobacco smoking was evaluated using the 6-item Fagerstrom Tolerance Scale (FTS) (30, 31). These participants were then divided into low (FTS score: 0–4) and medium to high (FTS score: 5–10) cigarette dependence groups. In addition, participants who responded “yes” to “Have you ever used electronic cigarettes?” and “Have you used any form of additive products (e.g., alcohol, betel nut, or other illicit drugs such as amphetamine or ketamine)?” were considered to be e-cigarette users and users of other substances, respectively. A participant had a high and low weekly allowance if they received <NT$200 and ≥NT$200 weekly, respectively (in May 2022, US$1 = approximately NT$29).

### Statistical analysis

The primary outcome was e-cigarette use. We also collected data on tobacco smoking status. We used the Chi-square and two-sample *t*-test to determine whether e-cigarette and tobacco smoking differed in their risk factors. The risk factors for e-cigarette use were determined using the adjusted odds ratio (aOR) with 95% confidence interval (CI) in hierarchically multiple logistic regressions. To compare the importance of differ predictors dimension, the area under the curve (AUC) was applied to represent the prediction power among various models. The predictive power of each of the three dimensions that the risk factors fell under was determined using the receiver operating characteristic curve, where a greater AUC indicates greater predictive power. All statistical analyses were performed using SAS software (version 9, SAS Institute, Cary, NC, United States).

## Results

Among the 1,289 enrolled participants, 803 (62.4%) were male, and the majority were aged 12–18 years (mean age: 15.6 years). Moreover, 525 (40.7%), 340 (26.4%), and 424 (32.9%) participants were from junior, senior, and vocational high schools, respectively.

Table 1 presents the distribution of the sociodemographic characteristics of the e-cigarette nonusers and users. We found that 9.3% of all participants had used e-cigarettes at least once. The adolescents were significantly more likely to use e-cigarettes if they were male (*p* < 0.0001), were significantly older (*p* = 0.0029), were in a vocational high school (*p* < 0.0001), had parents with an education level below senior high school (both *p* < 0.0001), were living with their grandparents or other relatives or were living in a single-parent household (*p* < 0.0001), had a low allowance (*p* = 0.0051), had used another substance (*p* < 0.0001), had smoked tobacco (*p* < 0.0001), had at least one family member who smoked (*p* = 0.0032), and had positive close friends to e-cigarette use (*p* < 0.0001).

**Table 1:** Personal characteristics, family environmental and substance use status between the participants with electronic cigarette use and those without in adolescents.

Variable	Electronic cigarette use				*p* value
	No		Yes		
	*n*	%	*n*	%	
**Personal characteristics**					
*Sex*					
Male	692	86.2	111	13.8	<0.0001
Female	455	94.2	28	5.8	
*Age*					
≦13 years old	166	94.9	9	5.1	0.0029
14 years old	175	89.7	20	10.3	
15 years old	199	92.6	16	7.4	
16 years old	234	89.3	28	10.7	
17 years old	221	86.7	34	13.3	
≧18 years old	154	82.8	32	17.2	
*School status*					
Junior high school	485	92.4	40	7.6	<0.0001
Senior high school	329	96.8	11	3.2	
Vocational high school	336	79.2	88	20.8	
**Family environmental status**					
*Education level of father*					
Below of senior high school	761	86.8	116	13.2	<0.0001
Senior high school or above	389	94.4	23	5.6	
*Education level of mother*					
Below of senior high school	722	86.9	109	13.1	<0.0001
Senior high school or above	428	93.4	30	6.6	
*Family structure*					
Living with parents	914	91.2	88	8.8	<0.0001
Living with single parent, grandparents or other relatives	236	82.2	51	17.8	
*Allowance, NT$*					
Less than 200 per week	542	86.7	83	13.3	0.0051
200 per week or more	608	91.6	56	8.4	
Family economic status, mean (SD)	5.73	2.02	5.40	1.95	0.0638
**Substance use status**					
*Other substance use*					
No	915	94.5	53	5.5	<0.0001
Yes	235	73.2	86	26.8	
*Tobacco smoking use*					
No	1,107	96.9	35	3.1	<0.0001
Yes	43	29.3	104	70.7	
*Smoking status of family members*					
No	607	91.7	55	8.3	0.0032
Yes	543	86.6	84	13.4	
*Close friends’ reactions to electronic cigarettes*					
No	640	98.0	13	2.0	<0.0001
Yes	434	77.5	126	22.5	

SD, standard deviation; NT$, New Taiwan dollars.

Four regression models were constructed to identify risk factors (Table 2). Model I included risk factors in the dimension of personal characteristics, specifically age, sex, and school status. Female students were less likely to use e-cigarettes than male students (odds ratio [OR] = 0.44; 95% CI 0.28–0.68, *p* = 0.0003). Compared with junior high school students, senior high school adolescents were significantly less likely to use e-cigarettes (OR = 0.25; 95% CI 0.10–0.57, *p* < 0.0001) and vocational high school students were nonsignificantly more likely to use e-cigarettes (OR = 1.75; 95% CI 0.90–3.41, *p* = 0.2254). Model I had high accuracy (AUC = 73.13%).

**Table 2:** Prediction and risk factors for electronic cigarette use in adolescents.

Variable	Model I			Model II			Model III			Model IV		
	OR	(95% CI)	*p* value	OR	(95% CI)	*p* value	OR	(95% CI)	*p* value	OR	(95% CI)	*p* value
**Personal characteristics**												
Age	1.19	(0.99–1.44)	0.0582	1.20	(0.99–1.43)	0.0648	1.08	(0.88–1.33)	0.4811	1.02	(0.78–1.34)	0.4752
**Sex**												
Male												
Female	0.44	(0.28–0.68)	0.0003	0.42	(0.27–0.66)	0.0002	0.44	(0.27–0.72)	0.0010	0.70	(0.30–1.63)	0.8910
**School status**												
Junior high school												
Vocational high school	1.75	(0.90–3.41)	0.2254	1.47	(0.75–2.89)	0.1576	1.42	(0.67–2.98)	0.3077	0.98	(0.37–2.61)	0.8652
Senior high school	0.25	(0.10–0.57)	<0.0001	0.25	(0.10–0.59)	<0.0001	0.32	(0.13–0.80)	0.0005	0.69	(0.23–2.11)	0.4119
**Family environmental status**												
*Education level of father*												
Senior high school or above												
Below of senior high school				1.45	(0. 83–2.52)	0.1930	1.17	(0.63–2.18)	0.6350	0.88	(0.40–1.90)	0.7384
*Education level of mother*												
Senior high school or above												
Below of senior high school				1.12	(0.67–1.87)	0.6632	1.28	(0.73–2.26)	0.3715	1.92	(0.93–3.97)	0.0782
**Family structure**												
Living with parents												
Living with single parent, grandparents or other relatives				1.95	(1.31–2.90)	0.0010	1.49	(0.96–2.27)	0.0712	1.64	(0.93–2.89)	0.0864
**Family economic status**				1. 03	(0.93–1.14)	0.6052	0.97	(0.87–1.08)	0.6083	0.86	(0.50–1.46)	0.5714
*Allowance*												
Less than 200 per week												
More than 200 per week				1.33	(0.91–1.96)	0.1336	1.22	(0.80–1.86)	0.3493	0.93	(0.81–1.07)	0.3159
**Substance use status**												
*Other substance use*												
No												
Yes							4.46	(2.94–6.77)	<0.0001	2.49	(1.45–4.25)	0.0009
*Smoking status of family members*												
No												
Yes							1.06	(0.70–1.62)	0.7788	0.99	(0.58–1.69)	0.9616
*Close friends’ reactions to electronic cigarette use*												
No												
Yes							7.27	(3.93–13.44)	<0.0001	5.61	(2.78–11.31)	<0.0001
*Tobacco smoking use^g^*												
No												
Yes										37.23	(21.00–66.01)	<0.0001
Lack of fit	0.7586			1.00			1.00			1.00		
AUC	73.13%			75.91%			86.68%			93.80%		

OR, odds ratio; CI, confidence interval; AUC, area under the receiver operating characteristic curve.

Model II included risk factors in the dimensions of family environment and personal characteristics. Family structure was significantly related to e-cigarette use (adjusted OR [aOR] = 1.95; 95% CI 1.31–2.90, *p* = 0.0010). Model II was slightly more accurate than Model I (AUC = 75.91%).

Model III included risk factors in all three dimensions. E-cigarette use was significantly related to close friends to e-cigarette use (aOR = 7.27; 95% CI 3.93–13.44, *p* < 0.0001) and the use of other substances (aOR = 4.46; 95% CI 2.94–6.77, *p* < 0.0001). Model III was much more accurate than Models I and II (AUC = 86.68%); this indicated that substance use status is a strong predictor of e-cigarette use among adolescents.

Model IV included only tobacco smoking as a covariate, and e-cigarette use was affected by tobacco smoking (aOR = 37.23; 95% CI 21.00–66.01, *p* < 0.0001). Model IV was the most accurate model (AUC = 93.80%). Overall, the use of other substances, close friends to e-cigarettes, and tobacco smoking were independent risk factors for e-cigarette use in adolescents.

We also evaluated the risk factors for tobacco use. The distributions of the sociodemographic characteristics of tobacco users and nonusers are displayed in Supplementary Table S1. In total, 11.4% of the participants had smoked tobacco cigarettes and 17.8% had medium to high cigarette dependence. The results for the predictive power of each dimension with regard to tobacco use are presented in Supplementary Table S2.

Models I to III (now with tobacco use as the dependent variable) had AUCs of 78.19, 80.00, and 89.10%, respectively, similar to their respective AUCs when e-cigarette use was the dependent variable. Model I indicated that tobacco users were more likely to be male (aOR = 3.82; 95% CI: 2.33–6.29) and significantly more likely to be in junior high school than in senior high school (aOR = 0.15; 95% CI 0.06–0.40, *p* < 0.0001). Model III indicated that tobacco users were significantly more likely to have used other substances (aOR = 5.48; 95% CI 3.60–12.84, *p* < 0.0001) and have close friends who react favourably to tobacco smoking (aOR = 6.90; 95% CI 3.70–12.84, *p* < 0.0001). Overall, the independent risk factors for tobacco smoking in adolescents were being male, using other substances, and having positive close friends to tobacco smoking.

Tobacco use (OR = 76.49; 95% CI: 0.06–0.40, *p* < 0.0001; Table 3) and tobacco smoking dependence (OR = 113.81; 95% CI: 26.50–488.82, *p* < 0.0001) were significantly and positively associated with e-cigarette use.

**Table 3:** Tobacco smoking use and dependence status affect electronic cigarette smoking behaviors in adolescents.

Variable	Electronic cigarette use				OR (95% CI)	*p* value
	No		Yes			
	*n*	%	*n*	%		
Tobacco smoking use						
No	1,107	93.5	35	6.5		< 0.0001
Yes	43	16.7	104	83.3	76.49 (46.89 to124.79)	
Tobacco smoking dependence*						
No	1,148	90.9	116	9.1		< 0.0001
Yes	2	7.1	23	92.9	113.81 (26.50 to 488.82)	

* Fagerstrom Tolerance Scale of 5–10 were considered a medium/high dependent score as a tobacco smoking dependence.

OR, odds ratio; CI, confidence interval.

## Discussion

To the best of our knowledge, this study is among few studies investigated the related factors that predict e-cigarette use behaviors among ethnic Chinese adolescents (24–26, 32, 33). A total of 9.3% of adolescent students in our study had used e-cigarettes. Tobacco use, the use of other substances, and positive close friends to e-cigarettes were independent risk factors for e-cigarette use in adolescents. The predictive accuracy of personal characteristics, family environment, and substance use status for e-cigarette use were 73.13, 75.91, and 93.80%, respectively. Furthermore, tobacco smoking dependence influenced e-cigarette use among adolescents.

The prevalence of adolescent e-cigarette usage varies with time, area, ethnicity, socioeconomic status, definition, and assessment tool. In our 2020 study, the prevalence of ever having used e-cigarettes among adolescent students in the Chiayi area of Taiwan was 9.3%. The Global Youth Tobacco Survey in Taiwan revealed that the prevalence of e-cigarette use in the previous 30 days among adolescents was 3.54% in 2017 (10). The percentages of adolescent students in Hong Kong who reported “ever having used” and “currently using” e-cigarettes in 2013 were 3.3 and 1.1%, respectively (33). In China, 3.1% of adolescents reported ever having used e-cigarettes in 2015, although only 0.5% of those adolescents reported current e-cigarette use (34). The prevalence of ever using e-cigarettes among adolescents in ethnic Chinese populations is generally lower than that among individuals in Western countries: 4.7% among German adolescent students (19), 12% among Hawaiian students (mean age: 14.6 years) (35), 16.0% among French high school students (36), 17.4% among Finnish students (12–18 years old) (15), and 23.5% among Polish students (15–19 years old) (37). Even in our results, the prevalence of e-cigarette use among ethnic Chinese adolescents remains lower than that that among global adolescents. Nevertheless, given the increase in use, identifying the factors that increase the risk of e-cigarette use is crucial for the early prevention of e-cigarette use among ethnic Chinese adolescents.

In our multivariate logistic regression analyses of risk factors for adolescent e-cigarette use, substance use status had a more influential effect than that of personal characteristics and family environment (Table 2). The independent risk factors for adolescent e-cigarette use were personal tobacco smoking, the use of other substances, and close friends to e-cigarette use. Tobacco smoking has been associated with an increased risk of e-cigarette use among adolescents. Our findings are consistent with other published studies (11, 23). Our study further revealed that personal tobacco smoking (OR = 37.23) was the most influential factor determining adolescent e-cigarette use.

Furthermore, tobacco use and tobacco smoking dependence were risk factors for e-cigarette use, and their ORs were approximately 76 and 114, respectively. The result indicates that tobacco smoking dependence can affect adolescent e-cigarette use. Studies have demonstrated that adolescent e-cigarette use is associated with the use of other substances, such as binge drinking and cannabis use (16, 38). Empirical studies have also demonstrated that using e-cigarettes at any point during adolescence is associated with the use of other substances, such as alcohol, tobacco from shisha, and marijuana (14, 39). These findings are consistent with our findings, suggesting that more attention should be focused on multiple substance use among adolescents. Additionally, research on e-cigarette use has indicated that university students are influenced by their family members to a far lower degree than by their close friends (18). This conclusion agrees with our finding that close friends’ attitudes to e-cigarette use are predictors of e-cigarette usage.

We report three findings regarding the risk factors of e-cigarette use and tobacco use among adolescents (Table 2; Supplementary Table S2). First, our models had high accuracy (AUC > 80%) in predicting e-cigarette and tobacco use among adolescents, with greater accuracy for e-cigarette use (AUC = 93.87% vs. 89.10%). Second, substance use status was a key risk factor for e-cigarette use and tobacco use among adolescents. Third, family environment had a stronger relationship with e-cigarette use than tobacco use, although this relationship was nonsignificant after other risk factors were adjusted for. This result indicates that interventions against e-cigarette use among adolescents should also focus on the adolescent’s family environment.

Our study has several limitations. First, the study was cross-sectional and was conducted in one geographic area. Nonetheless, our findings related to e-cigarette use and tobacco smoking are consistent with those of other studies in Taiwan (8–10, 18) Second, measurement bias might exist because the participants were asked the sensitive question about whether they had ever used e-cigarettes. Furthermore, because of the self-reported nature of the questionnaire, the respondents may have underestimated their e-cigarette use. To minimise this bias, we used anonymous questionnaires and barred school staff members from being present during the survey. Third, we could not determine that the dose–response of tobacco use affects e-cigarette use in adolescents because we did not count the quantity consumption of cigarettes (e.g., how many cigarettes did the participants smoke every day?) in our study. Nonetheless, we determined the qualitative effect of cigarette dependence e-cigarette use by dividing the participants by FTS score. Fourth, and finally, our study did not investigate the reasons for the adoption of e-cigarette use behaviors among adolescents.

## Conclusion

The present study analyses the predictive power of personal characteristics, family environment, and substance use status in relation to e-cigarette use among adolescents. Policymakers should pay attention to those who have used tobacco or other substances and to the attitudes of close friend groups towards e-cigarettes when developing smoking prevention and cessation strategies, in addition to regulating e-cigarette advertising and adolescent access to e-cigarettes (23, 40).

## Data availability statement

The original contributions presented in the study are included in the article/Supplementary material, further inquiries can be directed to the corresponding authors.

## Ethics statement

This study was approved by the Institutional Review Board of Dalin Tzu Chi Hospital, Buddhist Tzu Chi Medical Foundation, Taiwan (approval number: B10904015). Written informed consent was waived because we used an anonymous survey.

## Author contributions

P-IL, P-SH, and J-HT conceptualised and designed the study, conducted the statistical analyses, drafted the initial article, and reviewed and revised the article. M-NL and H-YC supervised the data collection, helped with the initial drafting of the article, and reviewed and revised the article. Y-HY, K-FW, and P-SH assisted with the statistical analyses and interpretation of data. All authors have approved the final article and agree to be accountable for all aspects of the work.
