# Supplementary material for: Prediction and potential risk factors for electronic cigarette use behaviors among adolescents: a pilot study in Chiayi, Taiwan
Source: Front Public Health. 2023 Jun 15;11:1140615. doi: 10.3389/fpubh.2023.1140615 (PMC10311257; doi:10.3389/fpubh.2023.1140615)
Supplement: Supplementary file 1 [file Table_1.pdf]

Appendix Table 1 Personal characteristics, family environmental and substance use status between the participants with tobacco smoking use and those without in adolescents.

Variable	Tobacco smoking use				p value
	No		Yes		
	n	%	n	%	
Personal Characteristics					
Sex					
Male	676	84.2	127	15.8	< 0.0001
Female	463	95.9	20	4.1	
Age					
≦13 years old	166	94.9	9	5.1	< 0.0001
14 years old	176	90.3	19	9.7	
15 years old	197	91.6	18	8.4	
16 years old	240	91.6	22	8.4	
17 years old	210	82.4	45	17.6	
≧18 years old	152	81.7	34	18.3	
School status					
Junior high school	486	92.6	39	7.4	< 0.0001
Senior high school	332	97.6	8	2.4	
Vocational high school	324	76.4	100	23.6	
Family Environment status					
Education level of father					
Below of senior high school	752	85.7	125	14.3	< 0.0001
Senior high school or above	390	94.7	22	5.3	
Education level of mother					
Below of senior high school	720	86.6	111	13.4	0.0023
Senior high school or above	422	92.1	36	7.9	
Family structure					
Living with parents	904	90.2	98	9.8	0.0001
Living with single parent 、grandparents or other relatives	238	82.9	49	17.1	
Allowance, NT$					
Less than 200 per week	539	86.0	88	14.0	0.0033
200 per week or more	605	91.1	59	8.9	
Family economic status, mean (SD)	5.72	2.01	5.50	1.97	0.2055
Substance Use status					
Other substance use					
No	913	94.3	55	5.7	< 0.0001
Yes	229	71.3	92	28.7	
Peers' reaction to tobacco smoking use					
No	680	98.1	13	1.9	< 0.0001
Yes	462	77.5	134	22.5	
Smoking status of family members					
No	602	90.9	60	9.1	0.0065
Yes	540	86.1	87	13.9	

Tobacco smoking abuse					
No	1142	93.9	74	6.1	< 0.0001
Yes	0	0.0	73	100.0	

SD, standard deviation; NT$, New Taiwan dollars.
