# Supplementary material for: Prediction and potential risk factors for electronic cigarette use behaviors among adolescents: a pilot study in Chiayi, Taiwan
Source: Front Public Health. 2023 Jun 15;11:1140615. doi: 10.3389/fpubh.2023.1140615 (PMC10311257; doi:10.3389/fpubh.2023.1140615)
Supplement: Supplementary file 2 [file Table_2.pdf]

**Appendix Table 2 Prediction and risk factors for electronic cigarette use in adolescents.**

| | Model I | | | Model II | | | Model III | | | Model IV | | |
|---|---|---|---|---|---|---|---|---|---|---|---|---|
| **Variable** | **OR** | **(95% CI)** | **p value** | **OR** | **(95% CI)** | **p value** | **OR** | **(95% CI)** | **p value** | **OR** | **(95% CI)** | **p value** |
| Personal Characteristics | | | | | | | | | | | | |
| Age | 1.19 | (0.99 to 1.44) | 0.0582 | 1.20 | (0.99 to 1.43) | 0.0648 | 1.08 | (0.88 to 1.33) | 0.4811 | 1.02 | (0.78 to 1.34) | 0.4752 |
| Sex | | | | | | | | | | | | |
| Female | | | | | | | | | | | | |
| Male | 0.44 | (0.28 to 0.68) | 0.0003 | 0.42 | (0.27 to 0.66) | 0.0002 | 0.44 | (0.27 to 0.72) | 0.0010 | 0.70 | (0.30 to 1.63) | 0.8910 |
| School status | | | | | | | | | | | | |
| Junior high school | | | | | | | | | | | | |
| Vocational high school | 1.75 | (0.90 to 3.41) | 0.2254 | 1.47 | (0.75 to 2.89) | 0.1576 | 1.42 | (0.67 to 2.98) | 0.3077 | 0.98 | (0.37 to 2.61) | 0.8652 |
| Senior high school | 0.25 | (0.10 to 0.57) | < 0.0001 | 0.25 | (0.10 to 0.59) | < 0.0001 | 0.32 | (0.13 to 0.80) | 0.0005 | 0.69 | (0.23 to 2.11) | 0.4119 |
| Family Environmental Status | | | | | | | | | | | | |
| Education level of father | | | | | | | | | | | | |
| Senior high school or above | | | | | | | | | | | | |
| Below of senior high school | | | | 1.45 | (0. 83 to 2.52) | 0.1930 | 1.17 | (0.63 to 2.18) | 0.6350 | 0.88 | (0.40 to 1.90) | 0.7384 |
| Education level of mother | | | | | | | | | | | | |
| Senior high school or above | | | | | | | | | | | | |
| Below of senior high school | | | | 1.12 | (0.67 to 1.87) | 0.6632 | 1.28 | (0.73 to 2.26) | 0.3715 | 1.92 | (0.93 to 3.97) | 0.0782 |
| Family structure | | | | | | | | | | | | |
| Living with parents | | | | | | | | | | | | |
| Living with single parent, grandparents or other relatives | | | | 1.95 | (1.31 to 2.90) | 0.0010 | 1.49 | (0.96 to 2.27) | 0.0712 | 1.64 | (0.93 to 2.89) | 0.0864 |
| Family economic status | | | | 1. 03 | (0.93 to 1.14 | 0.6052 | 0.97 | (0.87 to 1.08) | 0.6083 | 0.86 | (0.50 to 1.46) | 0.5714 |
| Allowance | | | | | | | | | | | | |
| Less than 200 per week | | | | | | | | | | | | |
| more than 200 per week | | | | 1.33 | (0.91 to 1.96) | 0.1336 | 1.22 | (0.80 to 1.86) | 0.3493 | 0.93 | (0.81 to 1.07) | 0.3159 |
| Substance Use Status | | | | | | | | | | | | |

| | | | | | | | | | | | | |
|---|---|---|---|---|---|---|---|---|---|---|---|---|
| Other substance Use | | | | | | | | | | | | |
| No | | | | | | | | | | | | |
| Yes | | | | | | | 4.46 | (2.94 to 6.77) | < 0.0001 | 2.49 | (1.45 to 4.25) | 0.0009 |
| Smoking status of family members | | | | | | | | | | | | |
| No | | | | | | | | | | | | |
| Yes | | | | | | | 1.06 | (0.70 to 1.62) | 0.7788 | 0.99 | (0.58 to 1.69) | 0.9616 |
| Close friends’ reactions to electronic cigarette use | | | | | | | | | | | | |
| No | | | | | | | | | | | | |
| Yes | | | | | | | 7.27 | (3.93 to 13.44) | < 0.0001 | 5.61 | (2.78 to 11.31) | < 0.0001 |
| Tobacco smoking use[g] | | | | | | | | | | | | |
| No | | | | | | | | | | | | |
| Yes | | | | | | | | | | 37.23 | (21.00 to 66.01) | < 0.0001 |
| Lack of fit | | 0.7586 | | | 1.00 | | | 1.00 | | | 1.00 | |
| AUC | | 73.13% | | | 75.91% | | | 86.68% | | | 93.80% | |

OR, odds ratio; CI, confidence interval; AUC, area under the receiver operating characteristic curve.
